# Management of type 2 diabetes with a treat-to-benefit approach improved long-term cardiovascular outcomes under routine care

**DOI:** 10.1186/s12933-022-01712-4

**Published:** 2022-12-09

**Authors:** Mario Luca Morieri, Enrico Longato, Barbara Di Camillo, Giovanni Sparacino, Angelo Avogaro, Gian Paolo Fadini

**Affiliations:** 1 Department of Medicine, University of Padova, 35100 Padua, Italy; 2 Department of Information Engineering, University of Padova, 35100 Padua, Italy; 3 Department of Medicine, University of Padova, Via Giustiniani 2, 35128 Padua, Italy

**Keywords:** Adherence, Appropriateness, Guidelines, Pharmacology, Observational

## Abstract

**Background:**

Results of cardiovascular outcome trials enabled a shift from “treat-to-target” to “treat-to-benefit” paradigm in the management of type 2 diabetes (T2D). However, studies validating such approach are limited. Here, we examined whether treatment according to international recommendations for the pharmacological management of T2D had an impact on long-term outcomes.

**Methods:**

This was an observational study conducted on outpatient data collected in 2008–2018 (i.e. prior to the “treat-to-benefit” shift). We defined 6 domains of treatment based on the ADA/EASD consensus covering all disease stages: first- and second-line treatment, intensification, use of insulin, cardioprotective, and weight-affecting drugs. At each visit, patients were included in Group 1 if at least one domain deviated from recommendation or in Group 2 if aligned with recommendations. We used Cox proportional hazard models with time-dependent co-variates or Cox marginal structural models (with inverse-probability of treatment weighing evaluated at each visit) to adjust for confounding factors and evaluate three outcomes: major adverse cardiovascular events (MACE), hospitalization for heart failure or cardiovascular mortality (HF-CVM), and all-cause mortality.

**Results:**

We included 5419 patients, on average 66-year old, 41% women, with a baseline diabetes duration of 7.6 years. Only 11.7% had pre-existing cardiovascular disease. During a median follow-up of 7.3 years, patients were seen 12 times at the clinic, and we recorded 1325 MACE, 1593 HF-CVM, and 917 deaths. By the end of the study, each patient spent on average 63.6% of time in Group 1. In the fully adjusted model, being always in Group 2 was associated with a 45% lower risk of MACE (HR 0.55; 95% C.I. 0.46–0.66; p < 0.0001) as compared to being in Group 1. The corresponding HF-CVM and mortality risk were similar (HR 0.56; 95%CI 0.47–0.66, p < 0.0001 and HR 0.56; 95% C.I. 0.45–0.70; p < 0.0001. respectively). Sensitivity analyses confirmed these results. No single domain individually explained the better outcome of Group 2, which remained significant in all subgroups.

**Conclusion:**

Managing patients with T2D according to a “treat-to-benefit” approach based international standards was associated with a lower risk of MACE, heart failure, and mortality. These data provide ex-post validation of the ADA/EASD treatment algorithm.

**Supplementary Information:**

The online version contains supplementary material available at 10.1186/s12933-022-01712-4.

## Background

Over the last decade, recommendations for selecting the most appropriate glucose-lowering medications (GLM) for the management of type 2 diabetes (T2D) have changed in view of the evolving evidence. The 2018 ADA/EASD consensus represented a paradigm shift from treat-to-target to treat-to-benefit [1] approach carried on in the latest 2019 and 2022 versions [2, 3]. This change was driven by results of cardiovascular outcome trials (CVOTs), showing the benefits of SGLT2i and GLP1-RAs on cardiovascular outcomes and extra-glycemic endpoints, such as body weight, blood pressure, and hypoglycemia risk [4–6]. Effectiveness of these interventions has been confirmed in real-world studies (RWS) [7–10]. However, CVOTs and RWS focused on the comparison between specific GLM and placebo or active comparators but did not evaluate whether the general treatment approach followed international recommendations. A few studies have previously assessed the impact of the overall quality of care on glycemic or metabolic traits. For example, Ceriello et al. previously reported that a quality of care summary score incorporating process and outcome measures was associated with variability of risk factors for complications of type 2 diabetes [11].

Guidelines and treatment algorithms propose specific recommendations, including priority for initiating the various GLMs [1, 12]. In fact, some statements of the ADA/EASD treatment algorithm are based on indirect extrapolations from RCTs or expert opinions. Yet, no trial or RWS was designed to test whether each GLM should be used as first, second, or more advanced line of therapy for T2D. The prognostic impact of adhering to recommended guidelines has been examined for lipid-lowering therapies [13] but, to our knowledge, no study so far has explored whether being aligned or deviating from the ADA/EASD recommendations on the management of T2D affected patient outcomes, including cardiovascular events and mortality. We believe the analysis of big data from routine care databases can address this question, challenging the recommendations or providing a useful post-hoc validation.

The aim of the present study was to evaluate whether alignment of treatment to the 2018 ADA/EASD recommendations, was associated with better outcomes in terms of major cardiovascular events (MACE), heart failure (HF), and overall mortality in patients with type 2 diabetes in a real-world setting.

## Methods

### Study design and population

This longitudinal retrospective study analyzed data on patients with type 2 diabetes routinely followed at the diabetes outpatient clinic of the University Hospital of Padua from 2008 to 2018. Detailed data on demographics, medical history, and medications were linked with administrative databases including hospital discharge codes and death certificates, as described before [14].

We retained only patients with at least 3 visits and at least 6 months of observation in the database. A schematic representation of entry visit, index date, follow-up time, and end-of-follow up is graphically represented in Additional file 1: Figure S1. The first available visit in the dataset between January 1st 2008 and June 1st 2018 was considered as the entry visit (visit 1). All subsequent visits were included in the evaluation of the exposure. To allow a minimum time between exposure and outcome, outcomes were ascertained after visit 3, which was considered the index date. The end of follow-up was September 1st 2018 (the last available date in the administrative database including death certificates and hospital discharge codes) or the last day the patient was present in the database (e.g. if a patient moved to another region) or the date of death, whichever occurred first. As patients accessed the clinic at least once a year, they were considered lost to follow-up and censored after two years without an outpatient visit or hospital admission.

Individualized patient’s target HbA1c level was calculated according to a previously defined algorithm, using five objective parameters: life expectancy, comorbidities, macro-vascular and advanced micro-vascular complications, risk of hypoglycemia from treatment, and disease duration [15].

### Definition of exposure

To evaluate whether treatment was aligned with a modern treatment approach, we identified six domains reflecting the main ADA/EASD recommendations on the management of hyperglycemia in patients with type 2 diabetes issued in 2018 [2], which was the first consensus document embracing a treat-to-benefit rather than a treat-to-target approach. These domains were selected to cover appropriateness of treatments in various stages of disease (Table 1). At each visit, we evaluated, as a binary variable, the following domains to establish whether treatment was aligned with recommendations. Domain 1 (metformin use) was met if first-line treatment included metformin, while it was not when any other combination of treatment not including metformin was used as first-line without having tried metformin before, except for patients with CKD. Domain 2 (intensification) was not met when there was a lack of treatment intensification (i.e., add-on or switch to a different regimen) with an HbA1c was above individualized target for two consecutive visits. Domain 3 (second-line treatment): was not met when sulfonylureas (SU) or insulin were used as second-line treatments. Domain 4 (insulin use) was not met in the presence of an inappropriate use of insulin, defined as initiation of insulin before GLP1-RAs and before metformin, or using bolus regimens before basal insulin. Domain 5 (use of cardioprotective drugs) was not met when cardio-protective treatments (SGLT2is and GLP1-RAs) were not prescribed to patients with previous cardiovascular events or revascularization (without having tried them before). Domain 6 (use of weight-affecting drugs) was not met when weight-increasing drugs (SU, insulin, glitazones) were used in patients with obesity (BMI > 30  kg/m^2^) prior to weight-neutral or weight-decreasing drugs (metformin, GLP1-RA, SGLT2i, DPP4i). All these 6 domains were evaluated at each visit in each patient.

**Table 1:** Domains

Domain	Description	Definition of deviation in the presence of these conditions:
Domain 1:c Metformin use	A first-line treatment not including metformin or any other combination of treatment not including metformin (without having tried metformin before and without known eGFR < 60 ml/min)	“number of diabetic medication/s > 0” AND “metformin use = 0” AND “prior use of metformin = 0” AND “history of eGFR < 60 = 0”
Domain 2: Treatment intensification	Lack of treatment intensification (add-on or switch to a different regimen) when HbA1c was above personalized target for two consecutive visits	“two consecutive visits with Hba1c > Hba1c-targets = 1 ” AND “changes in treatments*=0” (*defined as add-on or switch to a different regimen in that visit as compared to the previous one)
Domain 3: Second line treatments	Use of sulfonylureas or insulin as second-line treatments	“number of diabetic medication = 2” AND [“sulfonylureas use = 1” OR “insulin use = 1”] AND “prior history of DPP4i = 0” AND “prior history of GLP1-RAs = 0” AND “prior history of SGLT2i = 0”
Domain 4: Insulin use	Inappropriate use of Insulin (e.g., initiation before GLP1-RAs or with bolus regimen before basal)	[“insulin use = 1” AND “prior history of GLP1RA = 0” AND “Last Hba1c > 10% = 0”] OR [“insulin use = 1” AND “prior history of metformin = 0” AND “history of eGFR < 60 = 0”] OR [rapid-insulin use = 1” AND “history of basal-insulin = 0” AND “Last Hba1c > 10% = 0”]
Domain 5: Use of cardioprotective drugs [evaluated only on visits after year 2014]	Lack of cardio-protective treatments in patients with previous cardiovascular events or revascularization	[“history of CVD = 1” AND “GLP1-RAs use = 0” AND “SGLT2i use = 0” AND “prior history of GLP1-RAs = 0” AND “prior history of SGLT2i = 0”] OR [“history of CHF = 1” AND “SGLT2i use = 0” AND “prior history of SGLT2i = 0” AND “history of eGFR < 60 = 0”]
Domain 6: Use of weight beneficial-natural drugs	Use of weight-increasing drugs (SU or insulin) in obese subjects (BMI > 30 kg/m2) prior to weight-neutral or weight-decreasing drugs (metformin, GLP1-RAs, SGLT2i, DPP4i)	{“BMI > 30” AND [(“insulin use = 1” AND “last_HbA1c > 10%=0”) OR “sulfonylureas /glinides use = 1” OR “TZD use = 1”] AND “prior history of metformin = 0”} OR {“BMI > 30” AND [(“insulin use = 1” AND “last_HbA1c > 10%=0”) OR “sulfonylureas /glinides use = 1” OR “TZD use = 1”] AND [(“prior history of DPP4i = 0” AND “prior history of GLP1-RAs = 0”) OR “prior history of SGLT2i = 0”]}

Definition of the 6 domains used to evaluate the overall treatment prescription among patients with type 2 diabetes. The criteria including use or prior use of SGLT2i were assessed only on visits after 1st January 2015 (since prior of that visits the SGLT2i use was not widely available in Italy)

*CHF* congestive heart failure,* DPP4i* Dipeptidyl peptidase-4 inhibitor,* GLP1-RAs* Glucagon-like peptide-1 receptor agonists,* OTP* outdated treatment prescription,* SGLT2i* Sodium-glucose transport proteins-2,* TZD* thiazolidinedione

Patients were in Group 1 when at least one domain was not met (i.e. deviated from recommendations) or in Group 2 otherwise (i.e. when all domains were always met according to recommendations). We compared outcomes of patients in Group 2 versus Group 1. The proportion of time being in Group 2 (cumulative time aligned with recommendations) was evaluated as the ratio between the cumulative months being in Group 2 and the total follow-up time to that visit.

These domains were built according to 2018 ADA/EASD guidelines but can be considered valid also according to the most recent 2022 ADA/EASD guidelines [2, 3]. The main exception is that SGLT2i or GLP1RA can now be considered appropriate as first-line treatment before metformin for patients at high or very high risk of cardiovascular disease, including heart failure, or renal disease. However, when data for this study were collected (2008–2018), such practice was very uncommon and applying a similar domain would made all subjects become unaligned to the recommendation because most patients in tertiary-referral outpatient diabetes centers in Italy have very-high cardiovascular risk [16].

### Definition of outcomes

The primary outcome was occurrence of the 3-point major adverse cardiovascular events (MACE, defined as cardiovascular death, non-fatal myocardial infarction, or non-fatal stroke). Secondary outcomes were (i) a composite of hospitalization of heart failure and cardiovascular death (HF-CVM); (ii) all-cause mortality.

Occurrence of the outcomes was ascertained through hospital discharge codes (based on ICD-9) and death certificates (based on ICD-10) as reported in the administrative databases. MACE was defined in the presence of any of the following ICD-9 codes: 410.x (acute myocardial infarction) or 430, 431, 432.x, 433.x, 434.x, 436, (hemorrhagic or ischemic strokes), or death with the following ICD10 codes (I20-I25, I46). HF-CVM was defined in the presence of any of the following ICD-9 codes: 428.x or death with the following ICD10 codes (I20-I25, I46).

### Statistical analysis

Continuous data are presented as mean and standard deviation, whereas categorical variables are shown as percentage. Comparisons between patients in the two groups were performed using Student’s t test or chi squared tests, as appropriate. Cox regression models for time-dependent covariates were used to evaluate the association between Group 2 and MACE, HF-CVM (both including recurrent events), or all-cause mortality. We also used multivariable adjusted models with increasing complexity to account for possible “healthy users bias” due to patients in generally better health status (e.g. on first-line treatment, or without obesity or with HbA1c at target) being more likely to be in Group 2. Model 1 included age, sex, study entry year, diabetes duration, and the following time-varying covariates: presence of cardiovascular disease (myocardial infarction, ischemic myocardial disease, cardiac revascularization or stroke), diabetic kidney disease (defined by reduced eGFR below 60 ml/min/1.73 m^2^ and/or albuminuria), macrovascular disease (including history of cardiovascular events and clinical or subclinical peripheral artery disease, e.g., presence of ultrasound-detected carotid plaque), and microvascular disease (retinopathy, neuropathy, or nephropathy). Model 2 was similar to model 1 with the addition of the following time-varying variables: line of treatment, latest BMI and HbA1c values, other medications (antiplatelet, statins, other lipid-lowering treatments, RAS blockers, calcium channel blockers, beta-blockers, diuretics, oral anticoagulants), presence of obesity and severe diabetes decompensation (HbA1c levels higher than 10%; yes/no). Model 3 was similar to model 2 with the addition of the history of cancer, chronic obstructive pulmonary disease, systemic inflammatory disease, and ultrasound-documented hepatic steatosis. Sensitivity analyses stratifying on cumulative time being in Group 2 greater or less than 50% were conducted using the fully adjusted model (model 3). The analyses on HF-CVM included also history of baseline HF in all models. The impact of lifestyle (i.e. smoking habits, alcohol consumptions and physical activities) or and socio-demographic variables (i.e. level of instruction, citizenship, marriage status) on top of model 3 was evaluated in additional sensitivity analyses, since these information were available for a subset of patients.

We also stratified patients according to key baseline characteristics to evaluate whether the impact of exposure on the outcomes was affected by the patients’ clinical phenotype. We performed additional sensitivity analyses using Cox Marginal Structural Models (MSM, implemented via the SAS %MSM macro), by fitting a weighted pooled logistic model using inverse probability weights for treatment and censoring. Briefly, by an inverse-probability of treatment weighing evaluated at follow-up visits, these Cox MSMs allow for appropriate adjustment of confounding when there are time-dependent confounders that might themselves be affected by previous treatment or exposures, e.g. accounting for confounding by indication and healthy users biases [17–20].

The impact of each domain on the overall effect of Group 2 on MACE, HF-CVM, and mortality was tested by removing one domain at a time. This defined 6 alternative Group 2 definitions, each excluding one of the six domains. Then, the association of these alternative Group 2 definitions with outcomes was tested, and their estimates compared by Wald test to those obtained using the standard definition of Group 2.

Extraction of electronic medical records allowed complete collection of data on medication prescriptions (with no missing). According to study diagram described in Additional file 1: Figure S2, all subjects included in the analyses had information allowing complete evaluation of all domains and all covariates used in the different multivariable adjusted models (all models were tested on the same number of subjects). The only exception was the sensitivity analyses adjusted by lifestyle and socio-demographic information that were available only in a subset of individuals. Statistical analysis was performed with SAS and significance set to p < 0.05.

## Results

### Characteristics of the study cohort

After database linking and applying exclusions, we included 5419 patients, with 37,988 person-years of follow-up (Additional file 1: Figure S2). The median follow-up was 7.3 years (IQR 4.2–10.3), patients had a median of 12 (IQR 6–18) outpatient visits, with 870 patients (15.6%) being censored for lost to follow-up. Baseline clinical characteristics are described in Table 2. The population was representative of outpatients with type 2 diabetes in the Italian clinical practice, being on average 66-year old with 7.6 years of known diabetes duration at entry, 41.2% female. Baseline BMI was 29.2 kg/m^2^ and HbA1c was 7.2% (55 mmol/mol). Most patients had hypertension or dyslipidemia, and only 11.7% had a prior history of MACE.

**Table 2:** Patient characteristics

Characteristics	Entry Visit				Last Visit		
	All (n = 5419)	Group 1 (n = 3106)	Group 2 (n = 2313)	P	Group 1 (n = 3522)	Group 1 (n = 1897)	P
Year of visit	2008 (2008–2012)	2008 (2008–2010)	2010 (2008–2013)	< 0.0001	2018 (2015–2018)	2017 (2016–2018)	0.02
Age, years	66.1 ± 11.4	67.2 ± 11.1	64.6 ± 11.5	< 0.0001	72.8 ± 11.5	71.5 ± 12.0	0.0002
Female, n (%)	2206 (40.7%)	1250 (40.2%)	956 (41.3%)	0.42	1406 (39.9%)	800 (42.2%)	0.11
Diabetes duration, years	7.6 ± 8.4	10.0 ± 9.2	4.4 ± 6.0	< 0.0001	15.4 ± 10.0	11.0 ± 8.2	< 0.0001
HbA1c target mmol/mol	55 ± 4	57 ± 3	52 ± 3	< 0.0001	61 ± 4	55 ± 3	< 0.0001
HbA1c target (%)	7.2 ± 0.5	7.4 ± 0.4	6.9 ± 0.4	< 0.0001	7.7 ± 0.5	7.2 ± 0.4	< 0.0001
HbA1c mmol/mol	63 ± 14	68 ± 14	57 ± 12	< 0.0001	61 ± 10	50 ± 7	< 0.0001
HbA1c (%)	7.9 ± 1.7	8.4 ± 1.7	7.4 ± 1.6	< 0.0001	7.7 ± 1.3	6.7 ± 0.9	< 0.0001
BMI, kg/m2	29.2 ± 5.0	28.9 ± 5.0	29.5 ± 5.0	< 0.0001	28.7 ± 5.4	27.9 ± 4.7	< 0.0001
Systolic blood pressure	140.1 ± 19.9	141.0 ± 20.3	138.9 ± 19.4	0.0001	139.4 ± 21.2	139.2 ± 20.8	0.84
Diastolic blood Pressure	80.1 ± 10.5	79.2 ± 10.3	81.3 ± 10.6	< 0.0001	75.1 ± 11.1	76.9 ± 10.5	< 0.0001
Total cholesterol, mmol/l	4.92 ± 1.11	4.79 ± 1.08	5.09 ± 1.14	< 0.0001	4.17 ± 1.02	4.34 ± 0.94	< 0.0001
HDL cholesterol, mmol/l	1.29 ± 0.39	1.29 ± 0.40	1.28 ± 0.38	0.30	1.26 ± 0.37	1.35 ± 0.38	< 0.0001
Triglycerides, mmol/l	1.71 ± 1.38	1.66 ± 1.17	1.78 ± 1.62	0.009	1.48 ± 0.95	1.40 ± 2.23	0.17
LDL cholesterol, mmol/l	2.87 ± 0.91	2.77 ± 0.88	3.01 ± 0.94	< 0.0001	2.25 ± 0.83	2.38 ± 0.78	< 0.0001
ALT, IU/L	29.5 ± 23.5	28.6 ± 23.8	30.6 ± 23.0	0.01	22.7 ± 16.9	23.4 ± 16.8	0.21
eGFR ml/min/1.73 m2	77.7 ± 20.1	77.0 ± 20.2	78.7 ± 20.0	0.006	68.9 ± 24.6	74.3 ± 21.2	< 0.0001
Comorbidities and complications							
Hypertension	4318 (79.7%)	2533 (81.6%)	1785 (77.2%)	< 0.0001	3137 (89.1%)	1607 (84.7%)	< 0.0001
Dyslipidemia	3453 (63.7%)	1938 (62.4%)	1515 (65.5%)	0.019	2738 (77.7%)	1391 (73.3%)	0.0003
Macro-vascular disease	1421 (26.2%)	961 (30.9%)	460 (19.9%)	< 0.0001	2194 (62.3%)	838 (44.2%)	< 0.0001
Prior MACE	636 (11.7%)	443 (14.3%)	193 (8.3%)	< 0.0001	1227 (34.8%)	108 (5.7%)	< 0.0001
CAD	395 (7.3%)	279 (9.0%)	116 (5.0%)	< 0.0001	797 (22.6%)	75 (4.0%)	< 0.0001
Heart failure	231 (4.3%)	173 (5.6%)	58 (2.5%)	< 0.0001	658 (18.7%)	94 (5.0%)	< 0.0001
Micro-vascular disease	1755 (32.4%)	1220 (39.3%)	535 (23.1%)	< 0.0001	2326 (66.0%)	969 (51.1%)	< 0.0001
Diabetic kidney disease	1381 (25.5%)	901 (29.0%)	480 (20.8%)	< 0.0001	2069 (58.7%)	904 (47.7%)	< 0.0001
Albuminuria	850 (29.3%)	620 (33.9%)	230 (21.4%)	< 0.0001	1283 (42.2%)	480 (29.1%)	< 0.0001
Retinopathy	554 (28.4%)	471 (35.8%)	83 (13.0%)	< 0.0001	984 (37.5%)	253 (18.4%)	< 0.0001
Medications							
Line of treatments	1 (1–2)	2 (1–2)	1 (0–1)	< 0.0001	3 (2–4)	1 (1–3)	< 0.0001
Metformin	1174 (21.7%)	19 (0.6%)	1155 (49.9%)	< 0.0001	401 (11.4%)	776 (40.9%)	< 0.0001
Insulin	1537 (28.4%)	1513 (48.7%)	24 (1.0%)	< 0.0001	2036 (57.8%)	55 (2.9%)	< 0.0001
Sulfonylureas/glinides	1921 (35.4%)	1802 (58.0%)	119 (5.1%)	< 0.0001	969 (27.5%)	260 (13.7%)	< 0.0001
Thiazolidinediones	83 (1.5%)	47 (1.5%)	36 (1.6%)	0.90	47 (1.3%)	19 (1.0%)	< 0.0001
DDP4-inhibitors	150 (2.8%)	64 (2.1%)	86 (3.7%)	0.0002	600 (17.0%)	368 (19.4%)	0.29
GLP1-RAs	33 (0.6%)	12 (0.4%)	21 (0.9%)	0.015	122 (3.5%)	114 (6.0%)	0.03
SGLT2- inhibitors	5 (0.1%)	2 (0.1%)	3 (0.1%)	0.43	135 (3.8%)	50 (2.6%)	< 0.0001
Antiplatelet	2123 (39.2%)	1356 (43.7%)	767 (33.2%)	< 0.0001	2070 (58.8%)	865 (45.6%)	0.02
Statins	2218 (40.9%)	1306 (42.0%)	912 (39.4%)	0.053	2374 (67.4%)	1198 (63.2%)	< 0.0001
Lipid-low-treatment	2411 (44.5%)	1419 (45.7%)	992 (42.9%)	0.040	2519 (71.5%)	1270 (66.9%)	0.002
ACEi/ARBs	3066 (56.6%)	1827 (58.8%)	1239 (53.6%)	< 0.0001	2396 (68.0%)	1239 (65.3%)	0.001
Beta-blockers	1239 (22.9%)	749 (24.1%)	490 (21.2%)	0.01	1357 (38.5%)	529 (27.9%)	0.04
Calcium-channel block.	1302 (24.0%)	797 (25.7%)	505 (21.8%)	0.001	1184 (33.6%)	580 (30.6%)	< 0.0001
Diuretics	2134 (39.4%)	1299 (41.8%)	835 (36.1%)	< 0.0001	1949 (55.3%)	883 (46.5%)	0.02
Socio-demographic (n = 3594)							
Italian citizenship	3438 (95.7%)	2063 (95.4%)	1375 (96.1%)	0.31	2337 (95.3%)	1101 (96.5%)	0.09
Level of education							
Primary	1835 (51.1%)	1170 (54.1%)	665 (46.5%)	< 0.0001	1287 (52.5%)	548 (48.0%)	0.02
Secondary	1489 (41.4%)	848 (39.2%)	641 (44.8%)		995 (40.6%)	494 (43.3%)	
Tertiary	270 (7.5%)	145 (6.7%)	125 (8.7%)		171 (7.0%)	99 (8.7%)	
Domestic partnership	3310 (92.1%)	1979 (91.5%)	1331 (93.0%)	0.10	2262 (92.2%)	1048 (91.9%)	0.71
Lifestyles (n = 1459)							
Regular physical activity	557 (38.2%)	275 (34.8%)	282 (42.2%)	0.004	365 (37.3%)	192 (39.8%)	0.36
Regular alcohol consumption	563 (38.6%)	293 (37.0%)	270 (40.4%)	0.19	373 (38.2%)	190 (39.4)	0.64
Active smokers	233 (16%)	122 (15.4%)	111 (16.6%)	0.53	158 (16.2%)	75 (15.6%)	0.76

Clinical variables are shown for patients in Group 1 and Group 2 at entry visit or at the last visit before death or censoring

*BMI* Body mass index,* HDL* high-density-lipoprotein,* LDL* low-density-lipoprotein,* AST* aspartate transaminase,* ALT* alanine transaminase,* eGFR* estimated Glomerular Filtration Rate,* MACE* Major Adverse cardiovascular events,* CAD* Coronary Artery Disease,* DPP4* Dipeptidyl Peptidase-4,* GLP1-RAs* Glucagon-Like Peptide 1 Receptor Agonists,* SGLT2i* Sodium-glucose cotransporter 2,* ACEi/ARBs* Angiotensin-Converting Enzyme-inhibitors and Angiotensin Receptor Blockers. Domestic partnership (marriage or civil union)

At study entry (Additional file 1: Table S1), 57.3% (n = 3106) of patients were in Group 1 (i.e. had at least one unmet domain), whereas 42.7%; (n = 2313) fall into Group 2 (all domains met). By the end of the study (i.e. at the last available visit before death or censoring), 85.8% (n = 4649) had at least one visit in Group 1 due to one or more domains being unmet (Additional file 1: Table S1). By the end of the study, each patient spent on average 63.6% of time in Group 1 and 36.4% of time in Group 2. The domains most frequently unmet pertained to use of insulin (domain 4; 29.5% of time) and second-line treatments (domain 3; 26.5% of time). Not using drugs with cardiovascular protective effects was also highly prevalent (22.0% of time). As shown in Table 2, patients in Group 2 were younger, had shorter duration of diabetes, lower HbA1c, and lower prevalence of micro and macro-vascular complications as compared to those in Group 1.

### Risk of MACE

**Fig. 1:**
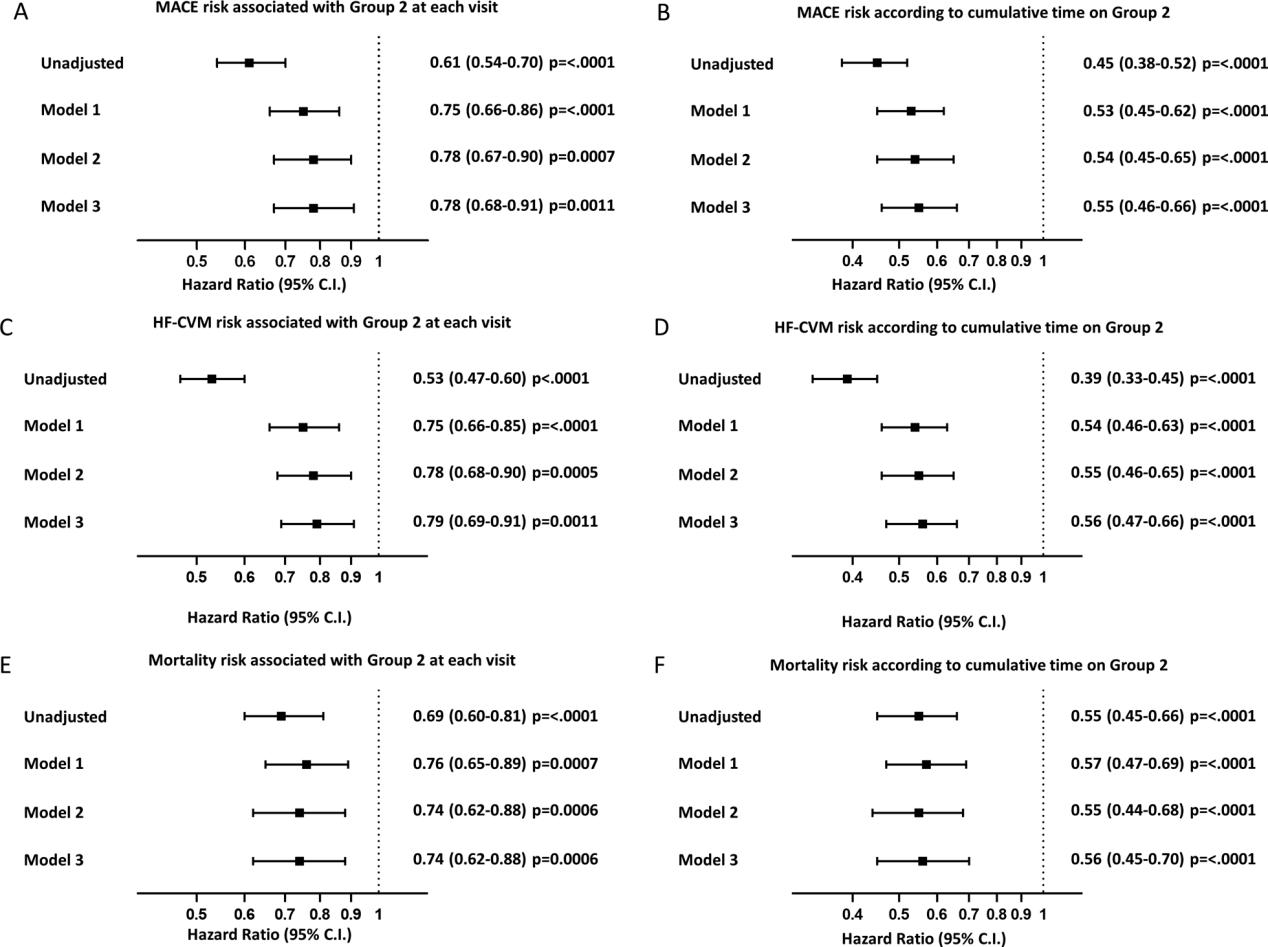
Outcome analysis. The association between Group 2 with MACE, HF-CVM and mortality is reported as forest plot. Panels **A**, **C**, **E** evaluated the risk associated with being in Group 2 at each visit, Panels **B**, **D** and **F** evaluated the risk according to the proportion of time being on Group 2. The plots show hazard ratios (HR) with 95% C.I. Number of subjects = 5419, MACE events = 1325, HF-CVM events = 1593, death events = 917

During follow-up, 1325 MACE occurred in 1117 subjects (20.6%), with an incidence rate to the first event of 31.0/1000 person-year. Group 2 vs. Group 1 was associated with a reduced risk of MACE in the unadjusted model (HR 0.61; 95% CI 0.54–0.70; *p* < 0.0001). This was confirmed, although slightly attenuated, in all models adjusting for possible confounders (Fig. 1A). In the fully adjusted model, at each visit, subjects in Group 2 had a 22% lower relative hazard of experiencing a MACE (HR 0.78, 95% CI 0.68–0.91; *p* = 0.0011). Figure 1B describes the estimated impact on MACE of cumulative time in Group 2 (i.e., the proportion of time being in Group 2 up to each visit, ranging from 0 to 1). The fully adjusted model (model 3) shows that being always in Group 2 (always free of deviations, i.e. cumulative time = 1) was associated with a 45% reduced hazard of MACE (HR 0.55, 95% CI 0.46–0.66; *p* < 0.0001) as compared to being always in Group 1 (i.e., cumulative time in Group 2 = 0).

In the first sensitivity analysis, we stratified cumulative time in Group 1 as a binary variable (i.e., low if the percentage of time in Group 1 was < 50% or high if the percentage of time was ≥ 50%). With this method, the association of Group 2 with reduced MACE incidence was confirmed (HR 0.67; 95% C.I. 0.58–0.78; *p* < 0.0001). The association with MACE was also confirmed in a second set of sensitivity analyses conducted in subsets of patients with further adjustment for lifestyle (1454 patients with 342 MACE events; HR for Group 2 vs. Group 1: 0.43, 95% C.I. 0.30–0.62, p < 0.0001) or socio-demographic characteristics (3594 patients with 1141 MACE events; HR: 0.61, 95% C.I. 0.50–0.74, p < 0.0001).

In a third sensitivity analysis using Cox MSMs models (weighted for the same covariates used in model 3), Group 2 was still associated with a 46% lower relative hazard of MACE (HR 0.64; 95% C.I. 0.56–0.72; *p* < 0.0001). In a fourth sensitivity analysis, we found that the significant association between Group 2 and lower MACE rate was confirmed when each single domain was individually excluded: no individual domain, when removed from the definition of Group 2, had major influences on the association with MACE (Additional file 1: Figure S3A).

### HF-CVM

We observed 1593 HF-CVM events in 1104 patients (multiple events being due to HF), with an incidence rate to first event of 30.5/1000 person-year. Group 2 was associated with a lower rate of HF-CVM, which was confirmed in all models. In the fully adjusted model, Group 2 was associated with a 21% lower hazard of HF-CVM (HR 0.79; 95% C.I. 0.69 to 0.91; *p* = 0.001; Fig. 1C). When cumulative time in Group 2 was analyzed (Fig. 1D), the fully adjusted model estimated that the relative hazard of HF-CVM was 44% lower (HR 0.56; 95% C.I. 0.47–0.66; *p* < 0.0001) for a patient being always in Group 2 as opposed to one being always on in Group 1. The association was confirmed when cumulative time in Group 2 was categorized (HR 0.69; 95% CI 0.60–0.80; *p* < 0.0001) and when the analyses were conducted in subsets of patients with further adjustment for lifestyle (1454 patients with 409 HF-CVM events; HR for cumulative time in Group 2 0.42, 95% C.I. 0.29–0.60, p < 0.0001) and socio-demographic factors (3594 patients with 1394 HF-CVM events; HR 0.64, 95% C.I. 0.53–0.77, p < 0.0001). Similarly, the Cox MSMs (weighted for the same covariates used in model 3) confirmed the association with an HR of 0.74 (95% C.I. 0.65–0.85; *p* < 0.0001). The association between Group 2 and HF-CVM was confirmed when each single domain was individually excluded from the Group definition (Additional file 1: Figure S3B).

### Overall mortality

917 patients died, equal to a mortality rate of 24.1/1000 person-year. Group 2 was associated with reduced mortality rates, which was confirmed in all models. In the fully adjusted model, Group 2 was associated with a 26% lower death rate (HR 0.74; 95% C.I. 0.62–0.88; *p* = 0.0006; Fig. 1E). When cumulative time in Group 2 was analyzed (Fig. 1F), the fully adjusted model estimated that the relative hazard of death was 44% lower (HR 0.56; 95% C.I. 0.45–0.70; *p* < 0.0001) for a patient being always in Group 2 as opposed to one being always on in Group 1. The association was confirmed when cumulative time in Group 2 was categorized (HR 0.67; 95% CI 0.56–0.81; *p* < 0.0001) and when the analyses were conducted in subsets of patients with further adjustment for lifestyle (1454 patients with 201 deaths; HR for cumulative time in Group 2 0.41, 95% C.I. 0.25–0.67, p = 0.0003) and socio-demographic factors (3594 patients with 733 deaths; HR 0.65; 95% C.I. 0.50–0.83, p = 0.0005). The Cox MSMs confirmed the association with an HR of 0.63 (95% C.I. 0.54–0.73; *p* < 0.0001). The association between Group 3 and mortality was confirmed when each single domain was individually excluded (Additional file 1: Figure S3C).

### Stratified analysis

**Fig. 2:**
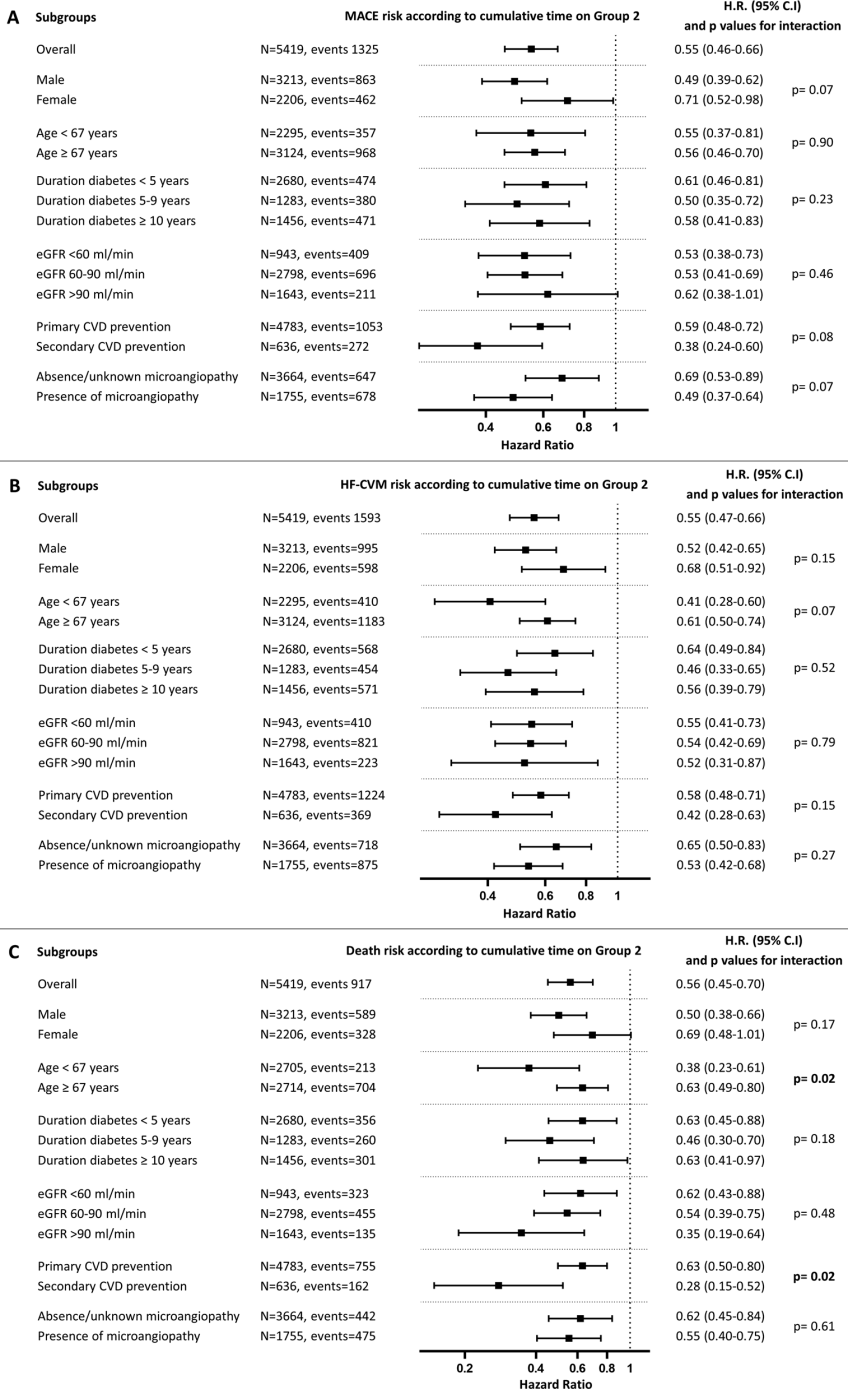
Subgroup analyses. The association between Group 2 and MACE (**A**), HF-CVM (**B**) or mortality (**C**) was evaluated within strata of the population defined by baseline clinical characteristics. The forest plot reports hazard ratios (HR) and the respective 95% C.I. with nominal p-values for the interaction

Stratifying the analysis by key clinical characteristics provided no evidence for a patient phenotype wherein the impact of being in Group 2 on MACE, HF-CVM, or mortality was lost. A nominally significant interaction was found between being in Group 2 and age on mortality, with more benefit in younger patients (Fig. 2). There was a nominally significant interaction between prior history of cardiovascular events and the effect of being in Group 2 on mortality, with a stronger effect in secondary cardiovascular prevention (Fig. 2).

## Discussion

In this study, we demonstrate that when therapeutic prescriptions for patients with T2D met internationally recommended standards, the rates of cardiovascular events and mortality were significantly lower.

Quality and quantity of the evidence supporting individualized therapeutic choices for the management of T2D have dramatically improved over the last 15 years [1, 2, 21–24]. However, most treatment algorithms and guidelines derive at least part of their recommendations by indirectly extrapolating results from trials or by consensus of experts. Indeed, though evidence-based, recommendations are to be considered opinions of the authors [1]. In the absence of enough evidence to support all statements, we believe it is critical to challenge or confirm the algorithm ex-post. Today, the wealth of information that accumulates within databases of routine clinical care can serve this purpose. We used the database of a large diabetes outpatient clinic, serving an urban area of ∼260,000 inhabitants in North-East Italy. The structure of the database has been described before, as clinical data routinely accumulated by Italian specialist diabetes clinics are stored uniformly in the same electronic chart and have been extensively analyzed for research purposes [11, 25, 26]. The dataset included more than 7,000 patients followed for more than 10 years, most of whom were seen more than 10 times at the same clinic, thereby building a solid basis to describe the patients’ trajectories and long-term outcomes. The population was representative of outpatients with type 2 diabetes seen in the specialist care setting [11, 27].

To define alignment or deviation from the international standards, we identified 6 domains from the 2018 ADA/EASD consensus [1] spanning several aspects of the pharmacologic management of T2D. Note that this approach was not meant to evaluate whether or not doctors followed recommendations available at the time. In fact, the 2018 consensus was issued after the data collection period (2008–2018). We reasoned that recommendations that are considered valid today would also have been valid in the past, under the assumption that their effect is not changing. As an exception, the domain pertaining to the use of cardio-protective drugs was assessed since 2015, when both GLP-1RAs and SGLT2is were available in Italy. We used the 2018 consensus because it represented a breakthrough paradigm shift. We did not incorporate the 2019 or 2022 update [2, 3] because the recommendation to use GLP-1RAs or SGLT2is in all patients with high cardiovascular risk independently from HbA1c would make most prior prescriptions inadequate and the study groups would collapse.

Among the study limitations, we acknowledge that the 6 domains were not exhaustive of the consensus and, to some extent, arbitrarily chosen. However, our sensitivity analysis suggests that no single domain individually explained the association with MACE and mortality. Therefore, it is arguable that the recommended therapeutic standard should be considered as a whole. In support of this view, whichever was the method for analyzing exposure (always vs. never in Group 2, or by cumulative time in Group 2, or categorized), the association with better outcomes remained similar. It should also be noted that domains may not be mutually exclusive. For example, not using metformin as first-line therapy (domain 1 unmet) would most likely draw a deviation on appropriate use of SU and insulin (domains 3 and 4 unmet), especially during years when fewer alternative GLM were available. While this may be perceived as a methodological limitation, it is the direct clinical consequence of patients proceeding along a therapeutic trajectory that would not be deemed appropriate according to current standards.

As another limitation, we recognize we had no information on treatment adherence, as pharmacy refill rates were not available in the database. Therefore, we only assessed whether prescriptions met domains of the 2018 ADA/EASD consensus, but not whether patients followed such prescriptions.

The lack of randomization remains an intrinsic limitation of this real-world study, leading to several confounding factors, measured and unmeasured, that are expected to influence the observed association between treatments and outcomes. While it is impossible to completely account for the effect of such confounders, we provide an extensive set of sensitivity analyses, with different modeling and adjustments, and all of them yielded consistent results even after accounting for possible confounding by indication or healthy user biases. Finally, these data are collected from a specialist outpatient service and generalizability to other settings, such as primary care, will require further studies.

The study has also notable strengths. Thanks to the large sample size and the high number of events recorded during long observation, all the analyses yielded robust estimates, with high statistical significance and precision. The effect was also stable across the spectrum of clinical characteristics as there was no stratum of the population wherein being in Group 2 (i.e. aligned with treatment recommendations) lost its significant association with better outcomes. Nonetheless, our analysis supports that deviating from the 2018 consensus exerted more detrimental effects in younger patients and in those who already had experienced a cardiovascular event. For these patients, strict adherence to treatment recommendations should be considered mandatory.

## Conclusion

In summary, we show that meeting few therapeutic recommendations for a modern management of T2D are significantly associated with lower risk of MACE, heart failure and premature mortality. These findings support ex-post validity of recent consensus and we argue that further studies should take the same path to validate treatment algorithms using routinely accumulated clinical data.

## Supplementary Information

**Additional file 1: Figure S1**. Schematic representation of study visits. The scheme shows visit for a hypothetical patient who accessed the clinic 12 times between Jan 2008 and Sept 2018 and how experienced two non-fatal MIs and finally died.** Figure S2.** Study flowchart. T2D, type 2 diabetes.** Figure S3.** Impact of individual domains. Attenuation of association with MACE (panel A), HF-CVM (panel B) and mortality (panel C) after exclusion of one domain at the time from the definition of overall inappropriate treatment prescription (ITP).** Table S1.** Prevalence of individual domains. Description of alignment with treatment recommendation on each domain at entry visit and during follow-up. §by design this domain required at least two visits to be evaluated. ^evaluated only on records from January 2015.

## Data Availability

The datasets used and analyzed during the current study are available from the corresponding author on reasonable request. Restrictions apply according to national and international regulations for personal data protection.
