# Supplementary material for: Management of type 2 diabetes with a treat-to-benefit approach improved long-term cardiovascular outcomes under routine care
Source: Cardiovasc Diabetol. 2022 Dec 9;21:274. doi: 10.1186/s12933-022-01712-4 (PMC9738028; doi:10.1186/s12933-022-01712-4)
Supplement: Supplementary file 1 — Additional file 1: Figure S1. Schematic representation of study visits. The scheme shows visit for a hypothetical patient who accessed the clinic 12 times between Jan 2008 and Sept 2018 and how experienced two non-fatal MIs and finally died. Figure S2. Study flowchart. T2D, type 2 diabetes. Figure S3. Impact of individual domains. Attenuation of association with MACE (panel A), HF-CVM (panel B) and mortality (panel C) after exclusion of one domain at the time from the definition of overall inappropriate treatment prescription (ITP). Table S1. Prevalence of individual domains. Description of alignment with treatment recommendation on each domain at entry visit and during follow-up. §by design this domain required at least two visits to be evaluated. ^evaluated only on records from January 2015. [file 12933_2022_1712_MOESM1_ESM.docx]

*Morieri et al.*

**Online Appendix**

**Figure S1. Schematic representation of study visits**. The scheme shows visit for a hypothetical patient who accessed the clinic 12 times between Jan 2008 and Sept 2018 and how experienced two non-fatal MIs and finally died.

**Figure S2. Study flowchart**. T2D, type 2 diabetes.

**Figure S3. Impact of individual domains**. Attenuation of association with MACE (panel A), HF-CVM (panel B) and mortality (panel C) after exclusion of one domain at the time from the definition of overall inappropriate treatment prescription (ITP).

**Table S1. Prevalence of individual domains.** Description of alignment with treatment recommendation on each domain at entry visit and during follow-up. Notes: §by design this domain required at least two visits to be evaluated. ^evaluated only on records from January 2015.

| **Domains** | **Entry Visit**  **% of deviations** | **Months from entry visit to first deviation*** | **Subjects with at least one visit with ITP during the entire study** | **% of time on deviation during the entire study** |
| --- | --- | --- | --- | --- |
| **D1: Metformin** | 1290 (23.8%) | 0.0 (0.0-0.0) | 1361 (25.1%) | 13.8% |
| **D2: Intensification of HbA1c control ^§^** | n.a. | 10.1 (4.8-23.9) | 3352 (61.9%) | 24.1% |
| **D3: 2^nd^ line treatment with SU/Insulin** | 1545 (28.5%) | 0.0 (0.0-17.8) | 2802 (51.7%) | 26.5% |
| **D4: Insulin Regimen** | 1510 (27.9%) | 0.0 (0.0-21.3) | 2437 (45.0%) | 29.5% |
| **D5: Cardio-renal protective drugs ^** | 87 (14.1%) | 81.3 (32.2-85.8) | 1079 (25.2%) | 22.0% |
| **D6: Weight neutral/benefit drug** | 939 (17.3%) | 0.0 (0.0-14.2) | 1716 (31.7%) | 19.0% |
| **Overall (all domains combined)** | 3106 (57.3%) | 0.0 (0.0-6.0) | 4649 (85.8%) | 63.6% |
